# Seal Whiskers Vibrate Over Broad Frequencies During Hydrodynamic Tracking

**DOI:** 10.1038/s41598-017-07676-w

**Published:** 2017-08-21

**Authors:** Christin T. Murphy, Colleen Reichmuth, William C. Eberhardt, Benton H. Calhoun, David A. Mann

**Affiliations:** 1 Naval Undersea Warfare Center Division Newport, 1176 Howell St., Newport, RI 02841 USA; 2 Woods Hole Oceanographic Institution, 266 Woods Hole Rd., Woods Hole, MA 02543 USA; 3 University of California, Santa Cruz, Institute of Marine Sciences, 115 McAllister Way, Santa Cruz, CA 95060 USA; 4 University of Virginia, Department of Mechanical and Aerospace Engineering, 351 McCormick Road PO Box 400743, Charlottesville, VA 22904 USA; 5 University of Virginia, Electrical and Computer Engineering, 351 McCormick Road PO Box 400743, Charlottesville, VA 22904 USA; 6 Loggerhead Instruments, 6576 Palmer Park Circle, Sarasota, FL 34238 USA

## Abstract

Although it is known that seals can use their whiskers (vibrissae) to extract relevant information from complex underwater flow fields, the underlying functioning of the system and the signals received by the sensors are poorly understood. Here we show that the vibrations of seal whiskers may provide information about hydrodynamic events and enable the sophisticated wake-tracking abilities of these animals. We developed a miniature accelerometer tag to study seal whisker movement *in situ*. We tested the ability of the tag to measure vibration in excised whiskers in a flume in response to laminar flow and disturbed flow. We then trained a seal to wear the tag and follow an underwater hydrodynamic trail to measure the whisker signals available to the seal. The results showed that whiskers vibrated at frequencies of 100–300 Hz, with a dynamic response. These measurements are the first to capture the incoming signals received by the vibrissae of a live seal and show that there are prominent signals at frequencies where the seal tactogram shows good sensitivity. Tapping into the mechanoreceptive interface between the animal and the environment may help to decipher the functional basis of this extraordinary hydrodynamic detection ability.

## Introduction

Seals use their whiskers to locate and follow hydrodynamic trails generated when objects move though the water. They can track biogenic and artificial wakes for several minutes after the disturbance has been generated^1, 2^, determine the direction of movement of the trail generator^3^, and discriminate between wakes produced by objects of different size and shape^4^. Scientific understanding of hydrodynamic receptor systems is limited compared to that of other sensory modalities, and mechanistic knowledge is lacking, especially at the level of signal reception^5, 6^. Many recent studies have focused on how the morphology of seal vibrissae suppresses vortex-induced vibrations (VIV), thereby reducing the noise generated by the structure when the animal swims through the water^7–9^. However, although the VIV are reduced, a distinct and rich vibrational signal is still elicited by the vibrissa. We hypothesized that these vibrations encode information about the characteristics of fluid flow fields and represent the crucial sensory input to the system.

**Figure 1:**
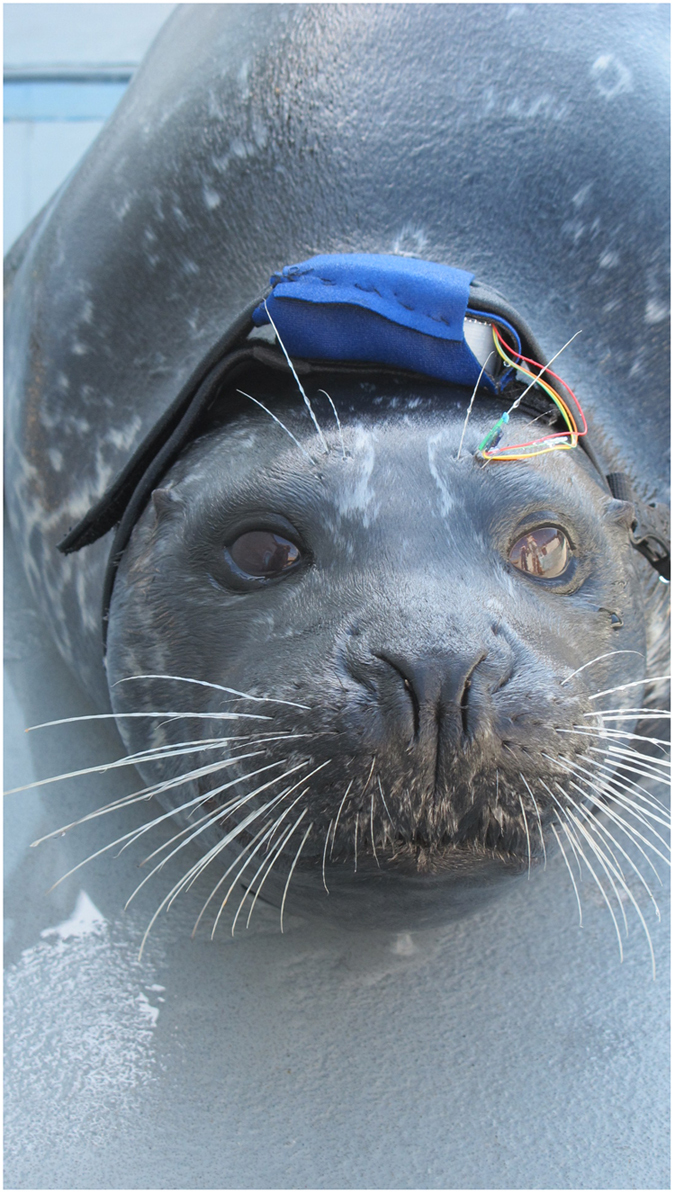
The accelerometer tag worn by a trained harbor seal. Photograph of the seal wearing the tag during live animal testing. The digital accelerometer is attached to a supraorbital whisker and connected by small wires to the datalogger, which is fitted to the animal’s head inside a pocket of the neoprene headband.

We recently reported that harbor seals are sensitive to mechanical vibrations from 10 Hz to 1,000 Hz^10^ – a range that extends much higher than the frequency content of hydrodynamic signals produced by swimming organisms. Here we show that seal whiskers vibrate within the sensitive range of the animal, and that these vibrations are altered by encounters with hydrodynamic disturbances. We introduce an animal-borne recording tag, which utilizes a miniature accelerometer (sampling at 600 Hz) affixed to the base of a seal’s whisker to directly record its motion. By harvesting the vibrations from the whisker during hydrodynamic tracking, we gain insight into the signals that are available to the sensory system. We directly measured the vibrational signals from excised whiskers in a flume tank as well as from a live harbor seal instrumented with the accelerometer tag (Fig. 1). In each set of tests, we measured whisker vibration in free-flow and disturbed flow at a low flow speed (0.5 m/s) and compared their spectral characteristics.

**Figure 2:**
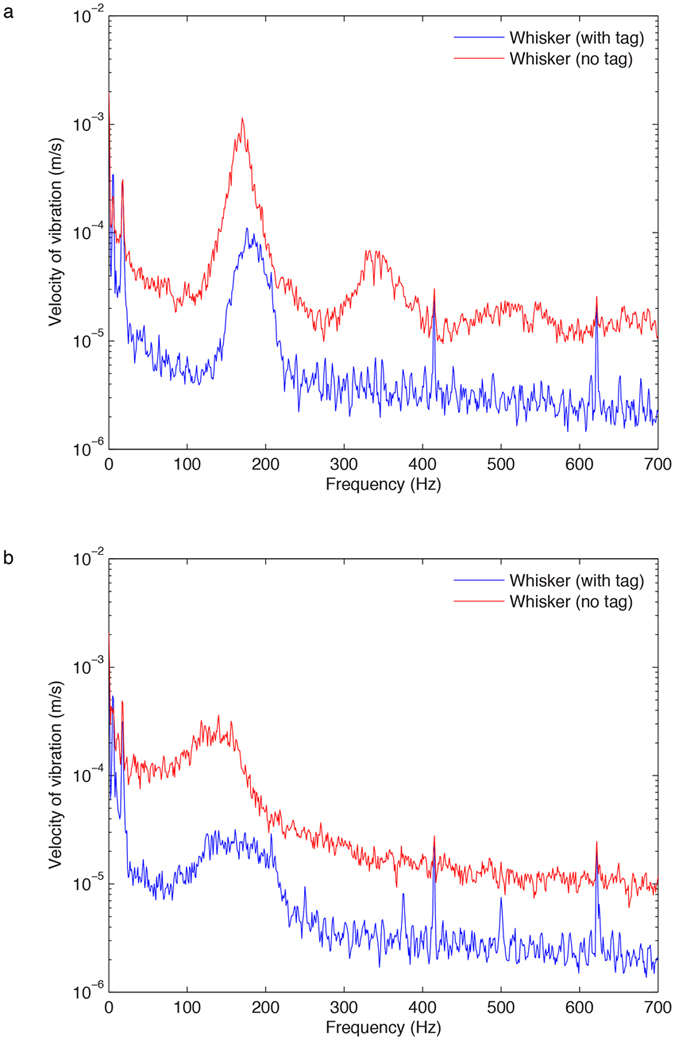
Overlaid velocity magnitude spectra for a representative recording of an excised whisker with and without the accelerometer, tested in a flume tank. (**a**) Vibration in *free-flow* condition. (**b**) Vibration in *disturbance* condition. Matching shapes of the spectra are seen between the whisker with and without the tag. This indicates that the attachment of the accelerometer caused some attenuation of the signal amplitude but the vibration pattern was still faithfully recorded.

Excised whisker testing in the flume tank utilized laser vibrometry and revealed distinct changes in vibration when the whisker encountered a hydrodynamic disturbance (Fig. 2). In *free-flow*, the whisker vibrated in a narrow frequency range. When a hydrodynamic disturbance from a cylinder with a shedding frequency of 4.2 Hz was placed upstream (*disturbance* condition), the vibrational signal spanned a broader range of frequencies. The difference between conditions can be quantified by comparing the Q value, a dimensionless parameter describing the bandwidth of a signal relative to its center frequency (Q_*free-flow*_ mean = 4.7, sd = 1.4; Q_*disturbance*_ mean = 1.4, sd = 0.5; p < 0.001). While the addition of a hydrodynamic disturbance caused the vibrations on the whisker to markedly shift from a narrowband to a broadband signal, it did not significantly change the overall energy of the whisker signal (p = 0.43).

Flume testing also allowed us to assess the effect of the tag on normal vibration of the whisker. When the laser vibrometer recordings of whiskers with and without the tag were compared, we found closely matched patterns in the vibrational signal (Fig. 2). The tag proved capable of recording changes in whisker vibration caused by a hydrodynamic disturbance under controlled conditions, as the presence of the tag did not affect the Q value (p = 0.32). There were no interaction effects of the presence of the tag and cylinder on the Q value (p = 0.13) or energy of the signal (p = 0.52). However, there was a significant broadband attenuation of the vibrational signal observed as an effect of the accelerometer attachment to the whisker (p = 0.004). While there is some mass loading from the accelerometer, the observed attenuation is likely due to the fact that the presence of the accelerometer changes the effective length of the whisker. Vortex shedding from a small part of the base will be altered by the accelerometer, however most of the whisker is still exposed and its vortex shedding will dominate the response. Therefore, the amplitude is reduced but the frequency pattern is maintained.

**Figure 3:**
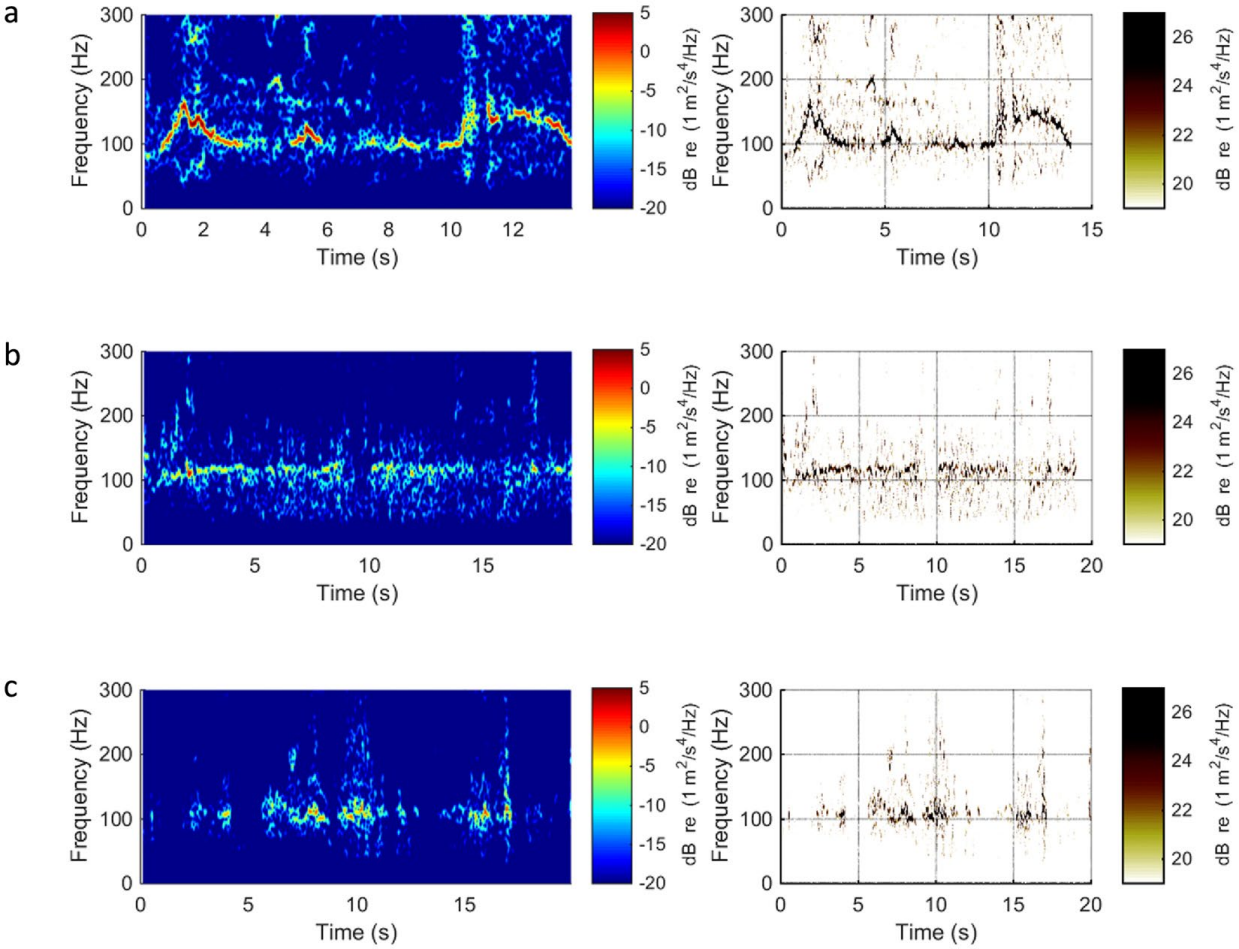
Tag recordings obtained from a live seal in the test pool under different hydrodynamic conditions. Spectrograms (left column) of whisker vibrations and reassignment method time-frequency representations (right column) for accelerometer recordings from the whisker of an actively swimming seal. Recordings are shown for different hydrodynamic conditions: (**a**) *Free-flow*: Animal freely swimming, (**b**) *Disturbance*: Animal following a sphere, and (**c**) *Disturbance*: Animal following radio-controlled model submarine.

During live animal testing, the accelerometer tag successfully recorded vibrations received from the supraorbital vibrissae of a freely swimming seal (Figs 3, S1–3). Similar to laboratory findings in the flume tank, a distinct vibrational signal is elicited by the vibrissa moving through the water. Differences between signal conditions in the live animal tests are noticeable but the trends are more difficult to observe than those in controlled laboratory conditions. In the free swim case, a strong narrowband signal is apparent. The dominant frequency in each time band fluctuates between 100 and 200 Hz, possibly changing with swim speed. The tag recordings for the two disturbance conditions show a more diffuse signal, and while it is difficult to quantify the spectral change, it is observable in the spectrograms that the energy content is spread across broader frequencies. The key point of these data is to show that whisker vibrations can be recorded *in situ* from a free-swimming seal and that they are relatively high frequency.

Some of the variability observed in the seal trials may be due to fluctuations in the animal’s swim speed or head turns while navigating in the circular pool. While efforts were made to maintain the animal at a steady speed, small variations were unavoidable with a live subject. While this complicates the comparison between experimental conditions in this study, it does highlight the fact that many aspects of the animal’s interaction with its environment are likely encoded in the whisker vibration, and present interesting topics for future investigation.

The observed patterns of whisker vibrations indicate that the signals on the whisker are dynamic and are affected by the hydrodynamic environment. The findings that seal whiskers vibrate in a narrow frequency band when moving through undisturbed water and that the frequency range of these vibrations broadens when exposed to hydrodynamic stimuli suggest how the receptor system might operate. If the vibration of the whisker shaft and deviations to the typical vibrational signal are detectable by the seal, they would provide salient information on the presence and nature of hydrodynamic stimuli. Recent psychophysical research on the vibrissal sensitivity of this species identified best sensitivity between 20 and 250 Hz and detectable signals up to 1000 Hz^10^. The signals recorded from the vibrissae in this study fall within the detectable range of the harbor seal, with much of the energy within the seal’s region of best sensitivity. Vibrations were observed up to 300 Hz in tag measurements, and higher frequencies would likely be captured with a higher sampling rate. Energy was recorded up to 600 Hz with the laser vibrometer in the disturbance condition, and we suspect that higher frequencies would be excited at increased speeds. The broad range of frequencies over which the whiskers vibrate in disturbed flow fields may explain why seals have detection abilities that extend beyond the low frequency region associated with biological hydrodynamic signals^5, 11^.

This is the first reported attempt to record and measure the sensory cues received by the whiskers of a live seal. Live animal and excised whisker testing of the accelerometer tag confirmed its ability to capture the signal received by an individual whisker with minimal effect on its natural vibration. The study provides a proof of concept that this approach may be utilized to gather *in situ* recordings from the vibrissae of a seal during hydrodynamic tracking. This method offers a glimpse of the sensory input for this complex system based on recordings from a single whisker and presents opportunity for continued investigation. Further research could explore signal characteristics associated with stimulus feature differences such as size and shape, as well as the relationship between disturbance-related vibration changes and those elicited by animal speed and whole-body movements. The recording system has the potential to be improved by hardware enhancements that accommodate multiple axis recordings at a high sampling rate, improved wiring design, and recordings at alternate points on the whisker array or multiple simultaneous recording points. Future iterations of this instrumentation might provide the opportunity to conduct field measurements on wild animals in order to investigate the function of the vibrissal system in a natural setting. While many data collection tools exist for flow measurement, the ability to do so at fine scale, on a moving platform, while operating in complex open-ocean environments is severely limited. Because the capability of the seal’s vibrissal system to detect and characterize hydrodynamic signals exceeds that of currently available technology^12^, examining the function of this sensory system in freely swimming animals can also aid the development of biomimetic systems.

## Methods

### Instrumentation

The accelerometer tag incorporates an Arduino-compatible datalogger connected to an external digital ADXL345 accelerometer (*Analog Devices, Norwood, MA, USA*) mounted to a PCB platform. The accelerometer and attached PCB surface mount board measures 7.0 × 4.0 × 1.0 mm and weighs 0.07 g. For attachment to the whisker, the external accelerometer was affixed with epoxy to a small section of flexible polyolefin shrink tubing and slid onto the whisker to be tested. In order to achieve a secure fit on the tapered form of the vibrissal shaft, the tubing was pre-shrunk on a sample whisker of comparable dimensions. During live animal testing, a small amount of synthetic rubber adhesive tack was applied to the attachment tubing in order to avoid slippage during active swimming.

The base of the datalogger was comprised of an OpenTag board (*Loggerhead Instruments*, *Sarasota*, *FL*, *USA*) and was powered by a 3.7 V 1300 mAh Li-Polymer cell rechargeable battery (product number 30108-1, *TENERGY, Freemont, CA*). For live animal testing, the datalogger was potted in epoxy (*MG Chemicals, Surrey, B.C*., *Canada*). When potted, the datalogger measured 5.4 cm × 3.3 cm × 1.8 cm and weighed 51 g. Recordings were made with a 600 Hz sample rate in the Z-axis of the accelerometer (direction normal to whisker and circuit board), and stored onto an 8 GB microSD card. Recordings were restricted to one accelerometer axis in order to accommodate the high sampling rate needed to capture the whisker vibrations. The direction of measurement is perpendicular to the long axis to the whisker, but is somewhat off angle to the rostro-caudal axis of the body due to the whisker’s orientation.

The accelerometer on the whisker was attached by four thin (32 gauge) wires to the datalogger. Because the accelerometer is digital, the signal quality is unaffected by wires connecting the external accelerometer to the datalogging board. For comfort of the animal, the section of wire between the accelerometer and datalogger was left unrestricted during live animal testing, and movement of the wires may have generated some noise. During excised whisker flume testing, the wires were secured to the sample holder to minimize movement. If future field deployments of the instrument are attempted, adhesives can be explored to fix the wires in the pelage hair of the animal. However, for this trained animal study, it was essential to make the device quickly removable and comfortable for the subject.

### Excised whisker flume testing

Laboratory testing in a water flume was conducted on nine excised mystacial whiskers (n = 9). Samples of closely matching lengths were selected to minimize variation (Mean length = 8.2 cm, s.d. = 1.2 cm). Samples were obtained from post-mortem stranded harbor seals (*Phoca vitulina*) at The Marine Mammal Center in Sausalito, California and stored in dry sample containers. Prior to testing, each specimen was rehydrated by immersion in fresh water for one hour. Rehydration as well as flume testing was conducted in fresh water due to constraints of the flume setup.

Experiments were conducted in a racetrack flume that was 76 cm wide, with a water depth of 25 cm. The flume had a paddlewheel propulsion system with flow straighteners that were upstream of the testing section. Vibrissal samples were held in the center of the water column in the transparent test section by attachment to a sting apparatus. The sting was composed of a stainless steel rod with a 90 degree bend and a streamlined plastic tip in which the whisker was mounted. The base of the whisker (approx. 0.5 mm) was inserted into the sting tip and firmly clamped in place by means of a recessed set screw. The clamped system was selected as a simplified model of the subdermal anchoring of the vibrissae but did not mimic the compliance of the tissue that exists in the natural system.

The body of the sting was positioned downstream of the sample and therefore did not interfere with the flow around the whisker. The whisker was mounted in the water column with its thin edge facing into the flow (0° angle of attack)^9^. The axis of measurement was perpendicular to the long axis of the whisker.

Laser vibrometry was used to measure motion of the excised whisker fitted with the accelerometer board and after the board was removed, without moving the whisker. Recordings were made with a laser vibrometer (CLV1000 controller with CLV700 sensor) (*Polytech Inc., Irvine, CA, USA*) placed outside the flume. This instrument measured point velocities on the whisker, from the position of the accelerometer (placed 12% up the vibrissal shaft) and from the identical point on the shaft, without the instrumentation. These points are referred to as *whisker (with tag)* and *whisker (no tag)*, respectivel*y*. Recordings were also taken from the sting mount to measure vibrations induced by the apparatus itself. In all circumstances, the laser was focused on the recording point, and vibrations in the cross-stream direction were recorded for 12 seconds with a 12,207 Hz sample rate, using a custom-written MATLAB program.

Recordings were made on the whisker with and without the tag, under two conditions termed *free-flow* and *disturbance*. In the *free-flow* condition, the sample was exposed to undisturbed, laminar water flow. In the *disturbance* condition, the sample was exposed to a hydrodynamic disturbance, generated by inserting a 2 cm diameter metal cylinder 0.5 m upstream of the sample. All tests were conducted at a flow speed of 0.5 m/s. Flow speed was selected based on constraints of the flume system. This represents a slow swimming speed for the harbor seal, but is still within the range of swimming velocity utilized by these animals. In addition 0.5 m/s matched the operating speed of the radio-controlled submarine utilized in pool tests, and therefore the approximate speed of the live seal during tracking tasks.

### Live animal testing

A trained adult male harbor seal, identified as *Sprouts* (NOA0001707) served as the subject for this study. This seal was captive-born and aged 24 years at the time of testing. Experiments were conducted in a 22,000 gal, 8 m diameter seawater test pool. This pool was surrounded by haul-out decks and was part of the animal’s living enclosure at Long Marine Laboratory in Santa Cruz, California.

The seal was gradually trained using standard operant conditioning methods and fish rewards to wear a soft neoprene headband with detachable blindfold and to follow the wake generated by a submerged object moving through the water. Hydrodynamic signals were generated using a sphere (6.7 cm diameter) on the end of an extension pole (2 cm diameter), as well as a radio-controlled model submarine (length 78 cm, height 21 cm, beam 18 cm, propeller size 5 cm, 12 V motor propulsion) (*model Neptune SB-1, Thunder Tiger Corp, Taichung, Taiwan*). Both objects were moved at approximately 0.5 m/s.

During test sessions, the datalogger was mounted on the seal’s head by placement in the pocket of a soft neoprene headband. The accelerometer was securely and temporarily affixed to one of the subject’s supraorbital vibrissae (Fig. 1) with the wires extending behind the vibrissae to the datalogger. Supraorbital vibrissae were used in these measurements for ease of attachment and behavioral constraints. During experimental sessions the animal located a hydrodynamic disturbance produced by a moving object and tracked the wake from an offset of approximately 0.5 m. In order to characterize the background signal on the whisker elicited by swimming motion, measurements were also obtained with the animal free swimming without addition of a hydrodynamic disturbance. In order to match the recorded vibrissal signal to known behavioral events, an overhead video feed was concurrently recorded with the tag measurements. The accelerometer was synchronized to the video by a pulse recorded on both instruments.

### Ethics statement

Live animal research was authorized under National Marine Fisheries Service (NMFS) permit 14535 and behavioral training and instrument testing were carried out in accordance with approved guidelines. All experimental protocols were approved by the Institutional Animal Care and Use Committee at UCSC.

### Data analysis

Signal processing of recordings was carried out in MATLAB (Mathworks, Inc.). The flume data (sample rate = 12207 Hz, record length = 12 s) was segmented into 125 ms segments, using 1525 points, which corresponds to a bin size of 8 Hz. A Hanning window was applied to each segment of the data and then a fast Fourier transform (FFT) was applied to each windowed segment. The signals were high pass filtered at 60 Hz to avoid signals from the sting apparatus used in flume testing. The low amplitude, low frequency noise generated by the sting apparatus was identified by control recordings on the surface of the sting. For each whisker, the 96 windowed FFT segments were averaged to reduce noise and Q, a dimensionless parameter describing the ratio of the center, or peak, frequency of a signal relative to its bandwidth, was calculated. A repeated measures ANOVA was used to test for statistically significant changes in Q and energy as a function of the presence of the tag and the introduction of a hydrodynamic disturbance.

For the live animal recordings (sample rate = 600 Hz), data were high pass filtered at 30 Hz. Spectrograms of frequency versus time were generated using the acceleration data from tag recordings with a 1.2 Hz frequency resolution and a 94% overlap to provide good quality visualization of the frequencies that the signal displayed through time. The reassignment method, a technique for sharpening the time-frequency representation of the signal by placing the maximum energy contribution of a windowed signal segment at the time-frequency location where the phase changes the least within the window, was applied post processing for improved visualization (Fig. 3, right column). The live animal recordings were not analyzed statistically because trials were conducted on a single animal and slightly varied in swim speed. They are included to show qualitatively the first signals ever recorded on the free-swimming seal whisker.

## Electronic supplementary material

S1

S2

S3

supplemental video legends
