# Supplementary material for: Seal Whiskers Vibrate Over Broad Frequencies During Hydrodynamic Tracking
Source: Sci Rep. 2017 Aug 21;7:8350. doi: 10.1038/s41598-017-07676-w (PMC5566400; doi:10.1038/s41598-017-07676-w)
Supplement: Supplementary file 4 — supplemental video legends [file 41598_2017_7676_MOESM4_ESM.pdf]

Seal Whiskers Vibrate Over Broad Frequencies During Hydrodynamic Tracking

Christin T. Murphy*[1,2], Colleen Reichmuth[3], William C. Eberhardt[4] , Benton H. Calhoun[5] and David A. Mann[6]

Supplementary Video Legends:

S1. Overhead video footage of the seal freely swimming
S2. Overhead video footage of the seal following the hydrodynamic disturbance from a sphere
S3. Overhead video footage of the seal following the hydrodynamic disturbance from a radio-controlled model submarine
